# Chiropractors in Multidisciplinary Teams: Enablers of Colocation Integration in GP-Led Primary Healthcare

**DOI:** 10.3390/healthcare12090926

**Published:** 2024-04-30

**Authors:** Shauna Dawn Fjaagesund, Wayne Graham, Evan Jones, Andrew Ladhams, Mark Sayers, Gary Campbell, Xiang-Yu Hou, Marius-Ionut Ungureanu, Florin Oprescu

**Affiliations:** 1 School of Health, University of the Sunshine Coast, Sippy Downs 4556, Australiamsayers@usc.edu.au (M.S.);; 2 Health Developments Corporation, Morayfield 4506, Australia; 3 School of Business and Creative Industries, University of the Sunshine Coast, Sippy Downs 4556, Australia; 4 Faculty of Medicine, University of Queensland, St. Lucia 4072, Australia; 5 Brain Treatment Centre Australia, Morayfield 4506, Australia; gary@braintreatmentcentre.com.au; 6 Centre for Health Research, University of Southern Queensland, Toowoomba 4350, Australia; janet.hou@unisq.edu.au; 7 Department of Public Health, Faculty of Political, Administrative and Communication Sciences, Babeș-Bolyai University, 400084 Cluj-Napoca, Romania; marius.ungureanu@publichealth.ro; 8 Center for Health Workforce Research and Policy, Faculty of Political, Administrative and Communication Sciences, Babeș-Bolyai University, 400084 Cluj-Napoca, Romania

**Keywords:** general practice, primary care, chiropractic care, health services integration, chiropractor

## Abstract

The aim of this study was to explore and document the enablers and barriers of chiropractic care colocation in general practice at a large-scale private primary care centre in Australia. This study focused on the perceptions of healthcare professionals regarding this integration. The research setting was a large integrated primary care centre located in an outer metro, low-socioeconomic area in the City of Moreton Bay, Queensland, Australia. Participant inclusion criteria included general medical practitioners, practice nurses, and medical managers who self-reported interactions with the physically collocated and integrated chiropractic practice. Data was collected from 22 participants using face-to-face, qualitative, semi-structured interviews with an average duration of 32 min. The data collected included perceptions of chiropractic treatment, enablers to patient referral pathways, and views of the integrated chiropractic care model. A reflexive thematic analysis was conducted on the data set. All participants reported that this was their first exposure to the colocation of a chiropractor within a general medical practice. Four key enablers of chiropractic care integration were identified: (1) the practitioner [chiropractor], (2) the organisation [general practice], (3) consumer flow, and (4) the environment [shared spaces and tenant ecosystem]. The chiropractic integration enhanced knowledge sharing and interprofessional trust among healthcare providers. The formal reporting of patient outcomes and understanding of the chiropractor’s scope of practice further enabled referrals to the service. Shared administrative and business processes, including patient records, booking systems, and clinical meetings, facilitated relationship development between the chiropractor and referring health providers. Colocation as part of a larger primary care centre created proximity and convenience for health providers in terms of interprofessional communication, and for patients, in terms of access to chiropractic services. Existing governance structures supported communication, professional education, and shared values related to the delivery of patient-centred care. Identified barriers included limited public funding for chiropractic services resulting in reduced access for patients of low-socioeconomic status. Additionally, scepticism or negativity towards the discipline of chiropractic care was identified as an initial barrier to refer patients. In most cases, this view towards the chiropractor was overcome by regular patient reporting of positive treatment outcomes to their GP, the delivery of education sessions by the chiropractor for the health providers, and the development of interprofessional trust between the chiropractor and referring health providers. This study provides preliminary evidence and a conceptual framework of factors influencing the successful integration of chiropractic care within an Australian large primary care centre. The data collected indicated that integration of chiropractic care into a primary care centre serving a low-socioeconomic region can be achieved with a high degree of health provider satisfaction.

## 1. Background

Chronic musculoskeletal pain is a common condition worldwide, with lower back pain (LBP) having the highest disability burden [1]. It is estimated that up to 80% of people will experience non-specific LBP within their lifetime [2,3,4]. LBP can cause substantial health and financial burdens [5,6,7]. In 2017, LBP was the leading cause of years lived with disability (YLDs) globally with the prevalence increasing by 52.7% from 1990 [7]. LBP is a leading cause of disability in Australia, affecting approximately 16% of the population at any point in time, with most (90–95%) diagnosed as non-specific LBP [8,9]. Nationwide, three in four people (74%) with reported back problems have one or more other chronic conditions [8].

Females, lower socioeconomic populations, and older people are at higher risk of experiencing chronic LBP [1,6,7]. Importantly, people living with chronic back pain have more complex needs [10,11] because LBP lasting for multiple months or years is often associated with social and psychological issues that cannot be addressed by physical treatment alone [5]. The challenge for health systems is that each patient can experience non-specific LBP differently; thus, unimodal therapies may not be the best choice. The literature suggests that individualised LBP treatment should be deployed to address the unique needs of each patient [12,13]. Additionally, multidisciplinary integrated care could provide LBP patients with a greater choice of treatments tailored to personal preferences [11,14,15]. This can include non-pharmacological treatments for chronic pain such as chiropractic care, as well as additional interventions to address social and psychological issues [11,16]. Previous Cochrane systematic reviews indicate that the common approaches to spinal manipulation treatment used by chiropractors to treat LBP are as effective or have similar results to other therapies [17,18], and combined approaches have demonstrated small improvements in medium-term acute and sub-acute pain and disability from LBP [4].

Conventionally, chiropractic care is delivered in a monodisciplinary environment separate from allopathic medical clinics or integrated primary care centres [19], which further contributes to the uncertainty and concern of the chiropractic professional from other health providers [20]. In Australia, most people with LBP will access a medical practitioner first, with only 19% seeking chiropractic care as a first point of contact [21]. The preference to seek the help of medical practitioners before chiropractors for LBP has been reported across multiple high-income countries [4].

In the United States and Canada, the availability of funded healthcare coordination for veterans has enabled chiropractors to be part of complementary and integrated primary care approaches [16,22]. There is a limited amount of literature available on successful chiropractic colocation integration in general practice within North America [23,24,25]. However, some studies have identified processes that support communication and professional development within multidisciplinary teams as an enabler of chiropractic integration [20,23]. Limited research suggests that integrated chiropractic primary care models provide effective treatment for low-socioeconomic status patients despite this population facing significant financial barriers to access [10,11]. 

Thus, the aim of this study was to explore and document the enablers of integration of a chiropractic practice and the referral pathways between medical doctors and chiropractors within a private primary care centre serving a low-socioeconomic area in the City of Moreton Bay, Queensland. Specifically, the authors examined the experiences of allopathic primary healthcare providers towards collocated chiropractic care services. This included exploring the considerations of health providers that were viewed as enablers or barriers to onsite chiropractic care.

## 2. Methods

The researchers used a pragmatic study approach guided by the principle of information power [26] and the Guba and Lincoln framework [27] to collect and analyse prospective qualitative data obtained from a 15,000 sqm integrated private primary care centre, serving approximately 200,000 patients, in a low-socioeconomic outer metro area of Moreton Bay, Queensland, Australia. The research setting was selected due to its novel model of integrated chiropractic practice within the general medicine practice [28]. Purposive sampling was used to approach eligible onsite volunteer health provider participants who were current primary care practitioners (general medical practitioner, practice nurse) or involved in existing organisational patient-facing or patient-flow processes (support worker, medical manager). Data on primary care health provider perceptions of chiropractic treatment, enablers to patient referral pathways, and views of the integrated chiropractic care model within the allopathic practice provided were collected using qualitative, semi-structured, open-ended interview questions, including two Likert scales for confidence and satisfaction of chiropractic care delivered.

Research recruitment resulted in 22 participants (Table 1) who met inclusion criteria (Appendix A) into the study (25 approached, 22 accepted, 3 declined). Recruitment continued until information power and redundancy was achieved within each timepoint [26]. The presented sample represented approximately 30% of the regular onsite health provider workforce actively involved in the general practice allopathic referral pathways. Participation was voluntary, and the research project information sheet and consent forms were provided in advance to health providers, with signed consent occurring prior to interview. Interview guides were pre-tested by three research team members and two onsite health providers. The included participants (*n* = 22) were provided semi-structured, open-ended interview questions to explore the unique enablers of integrated chiropractic care within the context of an existing model of integration and multidisciplinary collaboration and its established referral pathways between allopathic medical practitioners and the collocated chiropractors (Appendix B). 

Data collection occurred in 2021 (*n* = 11) and 2023 (*n* = 11) using two different interviewers. The purpose of using two timepoints was to assess if there were changes over time in health provider perceptions using two different interviewers. The time separation between timepoints was influenced by available research project funding. Face-to-face interviews were conducted onsite in private to protect anonymity. Neither interviewer had relationships with the health provider participants or the chiropractic practice at the time of data collection or analyses.

The interview duration lasted an average of 32 min. Transcribed data from 22 interviews was manually coded using reflexive thematic analysis to generate initial patterns and grouped themes [29,30], which demonstrated information power [26] and redundancy [30], using two coders who were also the interviewers across the two timepoints. The resulting themes were collaboratively discussed among members of the research team, providing an opportunity to revise and define themes. No distinguishable changes in perception were observed between 2021 and 2023; thus, it was deemed safe to aggregate the data sets for final analyses. 

Team members acknowledged, discussed, and documented their own varied personal attitudes towards chiropractic care and exposure to the setting and participants studied as part of the reflexive process. Additionally, the aggregated findings were reviewed by two general medical practitioners and a chiropractor for applicability and credibility of results, including transferability into clinical practice, with input communicated back to the research team for further consideration. 

This study was conducted in line with the University of the Sunshine Coast ethics approval #A191263.

## 3. Results

All participants interviewed within the integrated primary care centre (*n* = 22) reported interactions with the collocated chiropractic practice. Participants were diverse in experience and role(s) within the primary care centre (Table 1). The sample included general medical practitioners (*n* = 10), practice nurses (*n* = 4), medical specialists (*n* = 3), managers (*n* = 4), and support workers (*n* = 1) working within the specialties of general practice, urgent care (acute), and musculoskeletal and/or pain management (Table 1). The majority of the study participants (*n* = 13) possessed over a decade of clinical experience in general practice. Five participants reported between five and ten years of experience in the field, while four participants indicated they had less than five years of general practice experience. To further deidentify participants, unique specialties and individual years of work experience were reported as undisclosed within the aggregated results (Table 1). 

**Figure 1:**
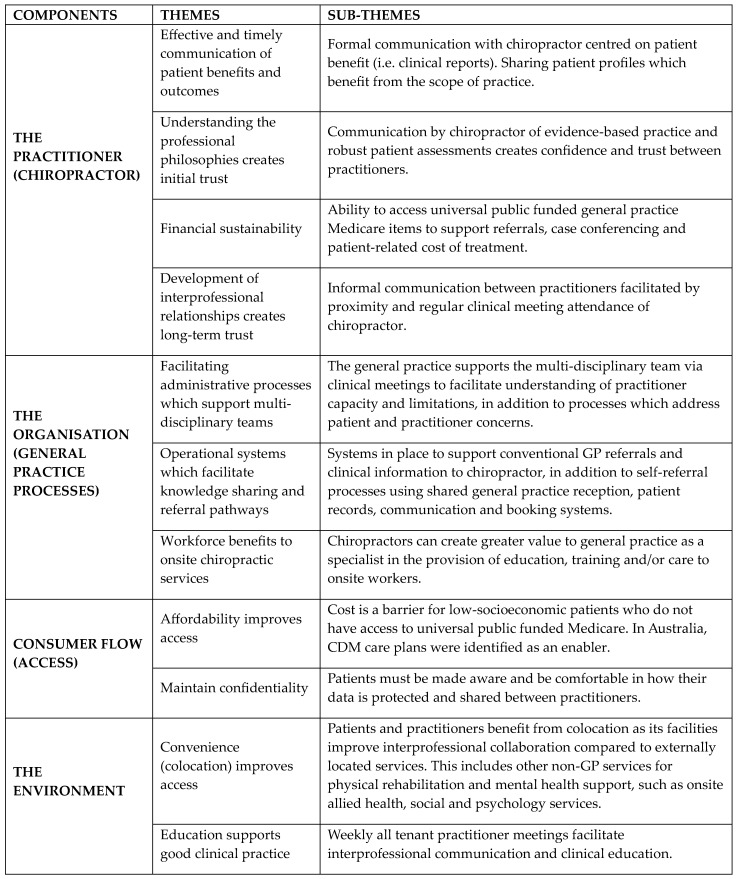
Four identified components of integrated chiropractic care in primary care from the thematic analysis.

Four identified components emerged from the thematic analysis of interview responses: (1) the practitioner, (2) the organisation, (3) consumer flow, and (4) the environment. The practitioner is described as the role of the chiropractor and the communication and understanding that was created at a practitioner-to-practitioner level. The organisation is explained as the general practice that embedded the chiropractor in its specialist area and facilitated processes for collaboration and referral pathways. Consumer flow refers to issues specific to health consumers, such as affordability of chiropractic services. The environment is described as shared spaces and the ecosystem of the greater primary care centre in which the chiropractor, general practice, and complementary health service tenancies operated. The practitioner had the most significant role in the creation of chiropractic care integration with the Organisation and Environment facilitating and supporting the share goals of collaboration and patient-centred care and Consumer Flow that generate the financial sustainability needed to justify the acceptance of the service (See Figure 1).

### 3.1. The Practitioner (Chiropractor)

#### 3.1.1. Communication of Patient Benefits from Chiropractic Care Improves Perception of the Discipline

Respondents (*n* = 12) reported that their perception of chiropractic care as a treatment modality improved with a more positive view (*n* = 12) after interacting and/or referring to the collocated chiropractor. Some participants did not report a change in their views (*n* = 9) towards chiropractic care and remained positive (*n* = 4), negative (*n* = 4), or neutral (*n* = 1) towards the discipline. No participants developed increased negative views towards chiropractic care or its discipline after working together within the general medical practice. Positive communication of patient benefits associated with the delivery of chiropractic care in the centre (*n* = 18) were described as important by participants to enable initial and continuing referrals. 

Participant #3: ‘Positive patient feedback is an enabler for referrals. It is impressive when asking a patient how a chiropractic visit went, and they had good outcomes’.

Participant #5: ‘My view towards chiropractors was always good [before working at the primary care centre], it is a bit higher now after meeting David as I have more respect for him and the profession…I would not refer to anyone I have not personally met [regardless of the profession]. It is best not to refer a patient to a practitioner you do not know. As if the patient has a poor outcome, it reflects [negatively] on you as a practitioner’.

Participant #9: ‘Before [working at] the hub [primary care centre] I did not refer to a chiropractor…I met David a few times and had good results with a patient and this helped enable me to refer to him…I refer 2 care plans per day at the hub which includes non-GP services. This is similar to what I did before the hub [with allied health] …plus now [I also can refer to] a chiropractor’.

Participant #14: ‘…one of the main reasons [for increased positive view] is that chiropractors are much more used here in Australia than where I used to work before [the primary integrated care centre]. And then you hear some good stories from patients, especially ones [patients] that benefit from chiropractic [care]’.

Participant #16: ‘I definitely feel like I encourage chiropractic care since starting here [primary care centre] than I did outside of my previous job’.

#### 3.1.2. Communication of Ideal Patient Profiles Opens Referral Pathways

Multiple participants (*n* = 10) indicated that it was important to understand which patient profiles were most likely to derive beneficial outcomes from the scope of chiropractic care offered. Matching patients to appropriate care was identified as a key consideration for referral to any treatment modality, including chiropractic care. Practitioners who referred patients to the chiropractic care (*n* = 14) to address pain also considered the patient’s willingness to engage in posture and strengthening exercises.

Participant #9: ‘Weekly lunch meetings are valuable as they let us know what kind of work David does and David explained to us the patient type, he accepts. Knowing this helps enable referrals to him’.

Participant #10: ‘Patient condition must match what the practitioner does. For example, physiotherapy is suited for strengthening of muscles. Chiropractor is better suited for patients who have posture-related issues…’

Participant #14: ‘…some patients really thrive from seeing a chiropractor and other patients don’t. So, you just adjust [referral] to what a patient has experienced before’.

Participant #22: ‘[I refer] posture related, occupational health\… [to chiropractor]. Basically, things which are non-surgical intervention, but generally [improve] by better posture adjustments. The patient needs to be the right mold…because David is…teaching people how to behave [improve posture]’.

#### 3.1.3. Understanding the Professional Philosophy of the Chiropractic Practitioner Creates Trust

Some participants (*n* = 8) indicated that the philosophy of the practitioner was important. Referring practitioners to the chiropractor described that alignment in philosophy was a key enabler to referral. Across interviews, this was described using a range of terms, including: evidence-based practice (*n* = 7), robust patient assessments (*n* = 4), confidence in treatment approaches (*n* = 3), published research (*n* = 3), and/or the inclusion of strengthening exercises (*n* = 3). Multiple participants (*n* = 4) who viewed evidence-based clinical practice as important also recognised that the onsite chiropractor held a PhD qualification and/or received his training in the United States. 

Participant #1: ‘David is exercise focused. This is big common ground for someone like me…I refer based on philosophy of chiropractor based on common goals to improve patient outcomes’.

Participant #18: ‘…We know chronic pain needs to be an active treatment whereby you are doing exercises, and you are strengthening weakened areas, you are changing biomechanics and posture. I like David because he does a combination of both [exercises and manual manipulation]’.

Participant #22: ‘As a doctor you are trained to look at evidence-based practice and it is often mentioned there is little evidence for chiropractic so that you are less likely to refer. I am happy to refer [patients] if they have a good experience with it [chiropractic care]’.

#### 3.1.4. Reporting and Communication of Patient Outcomes Is Expected

Based on feedback from patients who received treatments from the onsite chiropractor, most participants (*n* = 14) were highly satisfied or satisfied with the care provided (Appendix C). No participants were unsatisfied with the onsite chiropractor. The remaining participants who did not directly refer to the chiropractor cited role limitations (*n* = 6) or chose not to refer to any chiropractor (*n* = 2). Reporting was described as formal communication exchanged between the chiropractor and referring practitioner using a shared patient record. 

Participant #10: ‘I know David provides good service to patients as the feedback I receive from reports and the patients is excellent. Patients with realistic expectations and follow David’s strategy have good [high satisfaction] results’.

Participant #11: ‘Dr David demonstrated he does conduct assessments on patients before treating them. He provides reports and used an integrated care approach with patients. It is important doctors know as a referrer about patient outcomes’.

Participant #17: ‘I have no complaints from what I’ve heard [from patients]. I haven’t heard any bad reports that it was useless or anything like that. All the reports I’m getting back are that [chiropractic care] was worthwhile [for patient]’.

Participant #18: ‘The fact that [the chiropractor] works in this [integrated primary care] environment, the fact that he holds high regard in terms of our GP colleagues, and the fact that he we’re able to see his notes and he’s able to see ours [is the most influential factor to refer]’.

#### 3.1.5. Financial Sustainability of Chiropractor

Half of participants (*n* = 11) specifically identified the financial sustainability of the chiropractor as an important element of integration. These participants associated integration within a general practice with universal publicly funded care plans and reliance on patient referral pathways from general medical practitioners. Less participants (*n* = 3) mentioned concerns of poor public funding for chiropractic care. 

Participant #4: ‘There are not [appropriate] compensation measures to cover chiropractic…there is no rebate for GPs to refer to chiropractor. Case conferencing [renumeration] is limited… AHPRA [regulatory] restrictions on advertising testimonials and endorsements in adverts are not helping [chiropractors],’

Participant #6: ‘Everyone at the hub [primary care centre] needs a pathway for referral to reduce steps for patients to seek help. This includes on how non-general practice doctors and other tenants refer to David’.

Participant #15: ‘I think advantages [of chiropractic care onsite] would be that it would be a good way for the hub [primary care centre] to keep business in house. One stop shop…’

Participant #20: ‘…a positive is that Medicare [universal healthcare] does fund care plans for someone with a chronic condition. You are allowed five funded [no fee] appointments…so one or two appointments can be chiropractic’.

#### 3.1.6. The Development of Interprofessional Relationships Creates Trust

Participants more commonly referred to the chiropractor by first name (*n* = 12) or formally (*n* = 7), with a few (*n* = 4) using the term ‘the chiropractor’. Participants who referred to the chiropractor by first name (*n* = 12) also described interprofessional communication, beyond the expected patient outcome reporting, as important. This informal communication was facilitated by the convenience of colocation and/or social events, which brought practitioners together.

Participant #2: ‘Professional friendship and building connection with colleague is important’.

Participant #7: ‘Development of a relationship, trust and sharing of knowledge [with chiropractor] are enablers to referral’.

Participant #9: ‘Patient expectation is that you are sending them to a practitioner you know and trust’.

Participant #20: ‘[Chiropractors] are normally in a separate centre, and anyone here who works here says the greatest benefit is the congeniality and the integration and knowing everyone… [creating] a family setting’.

### 3.2. The Organisation (General Practice)

#### 3.2.1. Understanding Each Practitioner’s Scope of Practice Is Essential

It was identified by multiple participants (*n* = 9) that it was important to understand how the chiropractor’s scope of practice fits into existing treatment modalities. Specifically, referring practitioners (*n* = 5) and supporting roles (*n* = 4) within the organisation stated that it was important for chiropractors to be part of the multidisciplinary team to ensure that no one is practising unimodally or outside their capability and capacity. Chiropractic care integration was supported by existing organisational processes that invited the chiropractor to participate as a specialist.

Participant #6: ‘When a chiropractor is part of a multidisciplinary team there is advanced clinical governance, peer review and feedback. This does not happen in solo practice…’

Participant #10: ‘It is important that I know what the practitioner does, so that I can match the patient to the practitioner’.

Participant #18: ‘…we need to have clear boundaries about where they [chiropractors] fit. Some patients would only see GP’s [general medical practitioners] or only see chiropractors or only see nurse practitioners, so we just need to know [as referrers] when a case falls outside of their scope of practice, and this is central to having the GP sit in the centre of it [patient care]’.

#### 3.2.2. Organisational Processes That Support Referral Pathways

General medical practitioner referrals (*n* = 20) were described as the most common way to connect patients with onsite chiropractic care. Additionally, self-referral structures using specialist reception (*n* = 6), onsite conversation with a chiropractor (*n* = 6), and/or online booking systems (*n* = 2) were identified for patient or individual worker-initiated care. Some participants (*n* = 4) specified that shared internal communication and referral processes between practitioners onsite was superior to referring patients externally.

Participant #18: ‘…we can rub shoulders and because everyone inside the same building it is a much fluid and dynamic model. Outside the building you have to rely on the traditional methods of communication via fax or email and it just gets really disjointed’.

Participant #20: ‘Being able to book online, being able to book by phone and they [patients] can also call [the general practice] or speak to specialist reception…it is about making things easy for patients seeking care’.

#### 3.2.3. Health Provider Self-Care Benefits Related to the Integration of Chiropractic Care

Participants were asked if they have or would consider using chiropractic care themselves personally. Multiple participants (*n* = 8) had experiences with the onsite chiropractor practice as a patient or to pilot the patient assessment and treatment process. Many felt that they did not have a current health need for chiropractic care (*n* = 7), and if they did in the future, they would speak to the chiropractor directly (*n* = 4). Five participants deliberately adjusted their posture during interview, using a preventative technique taught by the chiropractor. Interviews also revealed that the onsite chiropractor provided guidance, advice, and/or treatment at no or low cost to onsite workers and health providers. Services provided by the chiropractor to the internal workforce were described as beneficial to own self-care (*n* = 3) and professionally helpful in better understanding treatment approaches (*n* = 5).

Participant #3: ‘Doctors seeing Dr. David as a patient helps them better understand the patient experience and the benefits [of chiropractic care] which would increase referrals’.

Participant #13: ‘…[it is important] to be inside the room as a patient and see what goes on [before you refer someone to a chiropractor]’.

Participant #18: ‘… I’m working close to the chiropractor, we work next door to each other…Talking to patients, hearing feedback from patients, talking to staff who actually use the chiropractor… internal feedback from staff is very positive …’

Participant #20: ‘…Dr. David had this poster he gave me, which gave me some exercises to improve my posture, show me how to sit in a chair properly and …I am using his posture exercises…this adds value to what he offers [the workplace]’.

### 3.3. Patient Access and Flow

#### 3.3.1. The Affordability of the Chiropractic Service Delivery Is Important

Multiple participants (*n* = 14) stated that chiropractic cost was a consideration for their patients, but not a barrier (*n* = 6). Health providers used enablers to reduce patient out-of-pocket costs, such as adding chiropractic services to universal publicly funded chronic disease management care plans (*n* = 12). Other views included that the patient must see value in chiropractic care to be willing to pay private out-of-pocket fees (*n* = 6), with trusted referring practitioners motivating patients to try non-pharmacological approaches when the health benefits outweigh the cost burden (*n* = 5).

Participant #2: ‘I can persuade most patients who can benefit from chiropractic care to pay, even if they are low income…. sometimes you have to just keep reminding patients about what is good for them each visit. There are always some [patients] who just want medicine for the pain and don’t want to do anything else [non-pharmacological]. If you have trust and rapport with patient, they will rely on your words even if there is a cost to treatment’.

Participant #7: ‘Patients with financial concerns can have chiropractic as part of their care plan’.

Participant #8: ‘…when there is a bill you have to ask the patient about their financial situation. You may need to dig a bit to see if cost is an issue…[in my own practice] …I do not have this cost discussion as I bulk-bill [no out-of-pocket fees]’.

Participant #9: ‘Yes, I use care plans to overcome financial concerns [out-of-pocket fees]. Many people cannot afford to continue going to more appointments over a long period of time’.

Participant #18: ‘…Dr. David is middle of the road when it comes to his costs [out-of-pocket fees]. My financial consideration [as a practitioner] is mainly about the longevity of treatment ….we are trying to be conservative with our billings and providing good, efficient quality medicine…’.

#### 3.3.2. The Sharing of Patient Health Information Enables Integration

Access to patient outcome information, including clinical notes and reports, were viewed as an important enabler for chiropractic referrals (*n* = 14). Some of these respondents (*n* = 6) specified the component of the sharing of patient record information as superior to other work locations or external referral pathways. Specifically, these participants described electronic communication exchange as convenient and efficient and a needed process enabler to integration with the chiropractor. However, some of these health providers (*n* = 3) that saw benefit to shared patient records also raised awareness of the responsibility that practitioners and practices have in maintaining patient confidentiality across multidisciplinary teams.

Participant #11: ‘Negatives of integration could include patient privacy issues ranging from (1) patients not wanting to release their health record [to the practice] and (2) some clinics not wanting external practitioners accessing their [clinical] notes’.

Participant #20: ‘…some tenancies have some level of patient record sharing…other tenancies don’t and there’s a multitude of reasons why that is…some of it is regulatory. With the chiropractor onsite [in the general practice] it is actually a positive [to share] as Dr. David can see the patient record and whatever the doctors have ordered and why. Dr. David sees [tests and clinical notes] before the patient comes into an appointment and knows his strategy in assessing as well as treatment for that patient’.

Participant #21: ‘…Software [is needed] to really take the next step into true integration…at the moment it’s very manual [for some tenancies] …the ability to [fully] share the data between all tenancies [would create] true integration’.

### 3.4. The Environment

#### 3.4.1. Convenience of Chiropractic Care Service Delivery (Colocation)

Most participants (*n* = 18) stated that there was an advantage to having a chiropractor within the same centre as it was convenient for both patient access and practitioners. The locality of an onsite chiropractor within the general practice was described by participants (*n* = 8) as an enabler of good interprofessional communication. Additionally, consistent availability of appointments (*n* = 6) was also an enabling factor for referral pathways to the chiropractor.

Participant #8: ‘Corridors permit [private] chit chat among colleagues, which is good. I enjoy the tearoom and having doctors knock on my door and ask for advice, even when I am busy’.

Participant #9: ‘Availability of appointments to book [enabler]. Wait times would be the opposite, a barrier. Patients will find someone else [another chiropractor or allied health] if they cannot easily get in. David is good as he doesn’t have long wait times’.

Participant #10: ‘Colloidal integration [physical] occurs at the hub [integrated primary care centre] which supports knowledge sharing and referrals’.

Participant #11: ‘Having the chiropractor here in the [integrated primary care centre] makes patients more open to use chiropractic care. By being here, there is more availability to see the chiropractor as a patient. By being here, doctors get to know the chiropractor which opens their minds too [to chiropractic referrals]’.

Participant #16: ‘… [patients] can literally be referred and then they can go straight to book an appointment [with chiropractor] …advising [patients] to go elsewhere [for care] …there could be disadvantages like [patient] compliance’.

Participant #18: ‘I think a lot of our patients here, travelling is difficult, they’re older, from a lower socio-economic background, funds are low, so to have an option of things onsite or very close’.

Most interviewed participants (*n* = 18) referred patients using traditional allopathic pathways. Many interviewed practitioners (*n* = 14) reported regular referrals, or as appropriate, to chiropractic care as an extension of non-pharmacological services available onsite. Across all non-GP, non-pharmacological services, physiotherapy (*n* = 16) was mentioned as the most frequent service referred to. Other common services referred to included: psychology or counselling (*n* = 11), exercise physiology or clinical group exercise (*n* = 8), occupational therapy (*n* = 7), and dietician or nutritionist (*n* = 6).

The knowledge of quality and service availability onsite was described by participants as an influence on individual referral patterns when compared with before and after working onsite. This included an increase in referral patterns to chiropractic care (*n* = 9), exercise physiology (*n* = 5), and addiction counselling (*n* = 3). A decrease in osteopath referrals (*n* = 5) was reported due to high cost to patient and/or service accessibility within the local area.

Two participants did not refer to the chiropractor unless a patient requested the service. These practitioners cited significant professional and/or personal past negative experiences with chiropractors before working at the primary care centre. Both participants (*n* = 2) had an unchanged towards the profession and low confidence in the services provided onsite (Appendix C).

Participant #1: We need more services like quality osteopath and remedial massage for patients to create a network for musculoskeletal support [onsite] for patients’.

Participant #14: ‘Most of my patients go to a physiotherapist, if I refer to an allied health professional…but there are others in the mix now, exercise [physiology] and chiropractor’.

#### 3.4.2. Education Supports Good Clinical Practice and Referral Pathways

The primary care centre hosts weekly clinical meetings for all onsite practitioners to participate in, including allied health. Participants who regularly attended these meetings (*n* = 11) described attendance and interactions with the chiropractor as an important enabler of referrals. Important elements within these meetings included clinical education sessions (*n* = 10), sharing of knowledge (*n* = 9), discussing referral pathways (*n* = 4), and building interprofessional relationships (*n* = 4).

Participant #2: ‘We come together so we can meet and talk to each other. When tenants have something to say, we remember them, and we don’t forget to refer. Even seeing a person in the room reminds you to refer…. It is about making connections [with chiropractor]’.

Participant #5: ‘Meeting David and having a chat at weekly clinical meetings [was enabler to refer]…he also did a presentation’.

Participant #6: ‘David has opportunity to engage and educate his colleagues and tenants in building at weekly clinical meetings’.

Participant #10: ‘Clinical integration tends to be impersonal. David teaching at a session makes it more personal and supports professional friendships’.

Participant #11: ‘Thursday clinical meetings and talking in the tearoom to Dr. David is important [referral enablers]’.

Participant #20: ‘Working in a in a collegial environment where you’re sharing information, going to clinical meetings, and learning together and becoming all better [as a practitioner] in your discipline is important’.

## 4. Discussion

The data collected and analysed indicated that integration of chiropractic care into a primary care centre serving a low-socioeconomic region can be achieved with a high degree of provider satisfaction (Appendix C). Overall, health providers reported that exposure to chiropractic care within the same location resulted in increased confidence in the quality of chiropractic care being delivered onsite (Appendix C). This aligns with existing literature focused on shared experiences and communication between health providers [23,31]. In presenting patient profiles where chiropractic care was viewed as beneficial, health providers recognised that their trusted relationship with patients encouraged the initial appointment. This aligns with previous literature that suggests trusted patient–provider relationships can influence treatment-hesitant patients to engage in chiropractic care [20].

Receiving positive patient feedback related to chiropractic treatment outcomes was viewed by most respondents as an important motivator of sending future referrals to chiropractic care. This aligned with previous literature focused on the reporting of positive patient outcomes [23]. In our study, patient feedback included communication of formal patient outcomes via reports from the chiropractor and self-reported patient outcomes. A shared electronic patient record [10] was highlighted by some respondents as an enabler of efficient communication, with the note that care must be taken to ensure that patient privacy is maintained [20].

A common facilitator across the respondents was the time invested by the chiropractor. This included building relationships and sharing knowledge with onsite providers. The study results suggest that health providers valued the development of interprofessional relationships and the sharing of information as enablers of quality of care. This aligns with previous research indicating that direct professional contact and clinical meeting attendance with individual chiropractors, focused on a common goal of sharing knowledge and professional development, was an integration and referral enabler [22,23]. Garner et al. [23] described the importance of chiropractor proximity with other providers in facilitating interprofessional contact and indicated that an experienced chiropractor (5+ years of practice) had a greater likelihood of integration success. 

Previous findings from Garner et al. [23] highlighted the importance of the chiropractor’s ability to communicate as an enabler to integration, specifically communication initiated by the chiropractor, facilitated by organisational processes, and encouraged by an ethical perspective by the greater primary care tenant environment. Additionally, data transcripts revealed that most providers used the chiropractor’s first name as opposed to his formal title or discipline, suggesting that close interprofessional relationships had been developed between both practices.

Understanding which types of patients would derive the most benefit from the chiropractor’s scope of practice was viewed as important in enabling appropriate patient referrals and increase positive patient outcomes. Many respondents identified that understanding the chiropractor’s scope of practice was key in the acceptance of its service delivery within the primary care centre. This aligns with Garner et al. [23], which suggested that increased knowledge of chiropractic care and practical experience of working within the same multidisciplinary team enabled successful integration.

Shared organisational processes were viewed as important by respondents from a patient management and interprofessional communication perspective. Many of the enabling processes identified were administrative and convenient for both patients and practitioners to use or access. For example, patient complaints were managed in collaboration with the chiropractor, general practice, and patient. The literature suggests that, in an ideal situation, the chiropractic discipline’s practice parameters would also be formalised within existing organisational clinical processes [20].

Participants described a philosophy of a patient-centred focus as a necessity for chiropractor acceptance within their organisation and a greater trusted referral network. This importance of evidence-based practice within collaborative multidisciplinary teams is supported in previous studies [15,31], with Mior et al. [20] specifically identifying the necessity of organisational alignment between chiropractic care and collaborative patient-centred values over an individual health provider’s personal or professional preference.

The financial cost of chiropractic care for the patients was identified as an important referral consideration and a barrier to care for patients from lower socioeconomic backgrounds. This aligned with previous studies that described chiropractic care costs as a barrier to patient access [20,23], particularly in low socioeconomic populations [10,11]. In Australia, at the time of data collection, there was limited universal public funding available for chiropractic treatment outside of short-term chronic disease management care plans or private health coverage.

Onsite chiropractic care costs for patients during the study period were viewed by health providers as reasonable and comparable with other services patients must pay for. These findings aligned with the idea that equity in reimbursement of services should be agreeable by all parties to enable collaboration within an integrated care service delivery model [20]. Health providers, reflecting on their own practice as business owners, viewed the financial sustainability of the chiropractor as an important consideration for integration. Adequate fee-for-service and consistent consumer flow from referrals was viewed as essential in the viability of chiropractic care integration within primary care. This included reference to the larger organisation generating consistent revenue, from multiple sources, to ensure that the chiropractor can provide quality service, an affordable fee structure, and the capacity to meet patient demand. The importance of renumeration structures and variability of profitability at fee-for-service sites were also noted as factors influencing the sustainability of integrated chiropractic care models [31].

Communication was described by health providers as both formal (patient reports, clinical meetings) and informal (interactions in shared spaces, weekly tenant lunch meetings). Shared processes of formal communication were facilitated by the organisation, whereas the environment created informal interactions through architectural and ecosystem design. A recurrent communication theme across health providers also included chiropractic education sessions and regular chiropractor attendance at clinical tenant lunch meetings. These onsite interactions were viewed as important in gaining organisational acceptance and in developing interprofessional trust to enable chiropractic referrals. This aligns with previous findings in which education sessions, location proximity, and attendance at team meetings were identified as enablers to chiropractic integration [15,20,23].

The location proximity of health providers, specifically within the general practice and specialist area of the primary care centre, provided physical convenience for medical doctors, nurses, and chiropractors to communicate and work in collaboration. This is supported by previous research that colocation is an enabler of collaboration in team service delivery [15,23]. Convenience was also described in that the integration of chiropractic care into a large-scale primary care centre provided greater accessibility and affordability to quality care, as it was in the same location as other needed health services. The affordability of chiropractic care was identified in previous literature as a benefit to low-socioeconomic populations [11] and older people [15].

Respondents also identified a unique cohort that benefited from the physical colocation and integration of chiropractic services. This included onsite health providers and workers who described both personal and professional benefits from delivered chiropractic care education, including preventative spine care behaviours and treatment approaches targeted at posture-related issues. This emergent theme was outside the scope of the study, but it suggests that there may also be a significant workforce benefit to onsite chiropractic care.

Some study limitations must be noted, including single-site data collection from one private, large-scale primary care setting in Australia located in an outer metro area. Changes in health provider perceptions between the two different timepoints was not observed. This may be attributed to the initial integration of the chiropractor occurring three years prior to the first data collection timepoint. Therefore, the results may not have analytical generalisability to high-density urban or low-population rural/remote areas. Additionally, the primary care centre studied had a high number of practitioners due to its increased patient load of servicing a high-needs and lower socioeconomic population. Unlike other countries, chiropractic care is not publicly funded outside of a limited number of appointments within a care plan. Therefore, the findings may not apply to smaller medical clinics, different patient population demographics, and/or other locations that have different health system funding models for chiropractic care. Site-specific tailoring and/or additional research are recommended points for implementation into other settings.

## 5. Conclusions

This study identified four key factors that influenced the successful implementation of integrative chiropractic care within a large-scale Australian primary healthcare centre. They are: (1) the practitioner (2), the organisation (3), consumer flow, and (4) the environment. These factors were not weighted in importance, but it is important to note the central role of the chiropractor in initiating relationship building. The chiropractor’s involvement in successful integration was undertaken by an experienced, evidence-based practitioner, with time investment in the provision of education sessions and the development of interprofessional relationships with onsite health providers. This investment in knowledge sharing, communication, and relationship building enabled the chiropractor to gain interprofessional trust and organisational acceptance to become part of the multidisciplinary primary care team.

Continued referrals to the chiropractic care were supported by general practice organisational communication, including interprofessional clinical education sessions and the sharing of patient profiles that fit the chiropractor’s scope of practice. Knowledge sharing included formal communication using shared organisational processes, which streamlined administrative activities, provided conventional allopathic referral pathways, and supported a collaborative environment focused on patient-centred care. The reporting of positive patient outcomes, either as self-reported outcomes and via shared patient records, were viewed as an important enabler of ongoing referrals to the chiropractor from medical doctors and nurses. The provision of accessible and affordable quality chiropractic care enabled consumer flow and demand for chiropractic services. Access to publicly funded services and/or fee structures in alignment to other similar services within the centre or area are needed to ensure financial sustainability of service delivery to low-socioeconomic populations. Adequate flow is an important integration consideration in private, fee-for-service settings to ensure quality care and capacity to meet demand.

Integration with the greater primary care centre provided the chiropractor convenience of proximity to develop interprofessional relationships with other health providers in addition to an opportunity to participate in facility-wide clinical governance. This supported deeper referral networks across a broader range of providers beyond the organisation, including allied health, psychologists, and specialists. The study findings may be transferrable within similar health systems and primary care centres in which service delivery is delivered by medical doctor and nurse referrals to chiropractors within a greater ecosystem support focused on collaboration between practitioners and patient-centred care. Site-specific tailoring and/or additional research are recommended points for implementation into other settings.

## Figures and Tables

**Table 1:** Summary of participant types within the integrated primary care centre collocated with the chiropractic practice.

Number	Participant Type	Sex	Period	Years Onsite ^1^	Specialty/Work Area ^1^
Data Collection Timepoint 1					
1	General Medical Practitioner	Male	2021 Q1	3+	General Practice/Specialty Undisclosed
2	General Medical Practitioner	Male	2021 Q1	3+	General Practice/Specialty Undisclosed
3	Nurse	Female	2021 Q4	3+	Urgent Care (Acute)/General Practice
4	General Medical Practitioner	Male	2021 Q4	1+	Urgent Care (Acute)/General Practice
5	General Medical Practitioner	Male	2021 Q4	1+	Urgent Care (Acute)/Specialty Undisclosed
6	Medical Specialist	Male	2021 Q4	1+	Urgent Care (Acute)/Specialty Undisclosed
7	General Medical Practitioner	Male	2021 Q4	4+	Urgent Care (Acute)/General Practice
8	Medical Specialist	Male	2021 Q4	3+	General Practice/Specialty Undisclosed
9	General Medical Practitioner	Male	2021 Q4	3+	General Practice/Specialty Undisclosed
10	Nurse	Female	2021 Q4	2+	Urgent Care (Acute)/General Practice
11	Medical Manager	Female	2021 Q4	2+	Specialty Undisclosed
Data Collection Timepoint 2					
12	Medical Specialist	Male	2021 Q4	2+	Specialty Undisclosed
13	General MedicalPractitioner	Male	2023 Q2	1+	Urgent Care (Acute)/General Practice
14	General Medical Practitioner	Male	2023 Q2	3+	General Practice/Specialty Undisclosed
15	Medical Manager	Female	2023 Q2	1+	Specialty Undisclosed
16	Nurse	Female	2023 Q3	4+	Urgent Care (Acute)/General Practice
17	General Medical Practitioner	Male	2023 Q2	1+	General Practice/Specialty Undisclosed
18	Nurse	Male	2023 Q2	3+	General Practice/Specialty Undisclosed
19	Nurse	Female	2023 Q2	3+	Urgent Care (Acute)/General Practice
20	Medical Manager	Female	2023 Q2	4+	Specialty Undisclosed
21	Medical Manager	Female	2023 Q3	1+	Specialty Undisclosed
22	General Medical Practitioner	Male	2023 Q3	4+	General Practice/Specialty Undisclosed

^1^ To protect anonymity of participants, individual fellowship completion, years of work experience, distinction between practice nurse/nurse practitioner and/or specific specialty are not specified in this table.

## Data Availability

The datasets presented in this article are not readily available due to healthcare data security reasons. Requests to access the datasets should be directed to sfjaages@usc.edu.au.

## Appendix A. Participant Inclusion Criteria

**Inclusion Criteria**
18 years of age or older
Exposure to the research setting as a current employee or contractor of the GP-led medical practice at time of data collection
Working role within the GP-led medical practice setting described as a doctor, nurse, manager or support worker
Received and read research project information sheet prior to interview
Provided formal written consent prior to interview
Re-reviewed research project information sheet and reaffirmed formal written consent verbally at time of interview
No withdrawal request was received pre, during or post interview
**Exclusion Criteria**
Working role was outside of a GP-led medical practice setting (i.e., pharmacist)

## Appendix B. Participant Interview Guide

**Interview Questions**
***Introduction: ask about participant about themselves to capture years of work experience, years onsite at Hub, current role***
**The next few questions relate to your perceptions of integration prior to and since working at the Hub**

1. What did service integration mean for you as a practitioner BEFORE working at the Hub?
  *Prompt: Infrastructure, Organisational Support, Clinical Support, Patient Support*
2. What does service integration mean for you as a practitioner AFTER working at the Hub?
  *Prompt: Infrastructure, Organisational Support, Clinical Support, Patient Support*
3. What is your experience with the integration of services AFTER working at the Hub?
  - Positive areas (advantages)
  - Negative areas (disadvantages)
  - Neutral areas (things you have seen and are not sure yet if it is a positive or a negative)

**The next few questions relate to your perceptions of chiropractic care *prior to* you working at the Hub**

1. Please describe what were your beliefs/perceptions about chiropractic care as a treatment option for your patients BEFORE working at the Hub.
2. Please list the types of non-GP services that you have referred to during your general practice experience BEFORE working at the Hub.
  *Prompt: If no, ask why (i.e., if role permits them to refer patients to services).*
  *Prompt: If no, ask how they assisted patients, family and/or friends to services onsite.*
3. From the list generated (question 5—BEFORE), please indicate the approximate average percentage of referrals.
  *Prompt: Please describe your frequency of referrals to non-GP services.*
  *Prompt: Were any of these services to a chiropractor? Why or why not?*
  *Prompt: What would have made it easier to refer/wayfind to a chiropractor?*

**The next few questions relate to your perceptions of chiropractic care *since* working at the Hub**

1. How (if at all) have your perceptions about chiropractic care as a treatment option for your patients changed AFTER starting your work at Hub?
  *Prompt: Changed (improved or worsened), Unchanged*
  *Prompt: Are your perceptions more ore remain Positive, Negative or Neutral*
2. Please list the types of non-GP services that you have referred to AFTER starting work at the Hub?
  *Prompt: If no, ask why (i.e., if role permits them to refer patients to services).*
  *Prompt: If no, ask how they assisted patients, family and/or friends to services onsite.*
3. From the list generated (question 2 – AFTER), please indicate the approximate average percentage of referrals
  *Prompt: Please describe your frequency of referrals to non-GP services.*
  *Prompt: Were any of these services to a chiropractor? Why or why not?*
  *Prompt: What would have made it easier to refer to a chiropractor?*

If chiropractic referrals were mentioned in question 3, describe the most influential factors that contributed to your decision to refer to the chiropractic services available at the Hub

4. Please list the main barriers that would prevent you from referring your patients for chiropractic care.
  *Prompt: Infrastructure, Organisational Support, Clinical Support, Patient Support*
5. What do you believe are the main enablers that would likely influence your decision to refer your patients for chiropractic care?
  *Prompt: Infrastructure, Organisational Support, Clinical Support, Patient Support*
6. What do you think would be the advantages (or disadvantages) of integrating chiropractic care into mainstream primary and allied health care facilities such as the Hub?
  *Prompt: What are the advantages of chiropractic integration at the Hub?*
  *Prompt: What are the disadvantages of chiropractic integration at the Hub?*
7. Are there any financial considerations associated with referring your patients to chiropractic treatment at the Hub?
  *Prompt: If financial barrier is mentioned and an enabler is not explained, ask if there is a way to overcome it.*
8. Please provide any comments about your confidence levels associated with chiropractic referrals at the Hub
  *Prompt: If given the choices of: Very Confident, Confident or Not Confident, which would you pick to describe the confidence levels associated with chiropractic referrals at the Hub?*
  *Prompt: Why is that?*
9. Based on any feedback from patients who have received chiropractic treatment, describe your overall level of satisfaction with chiropractic care at the Hub.
  *Prompt: If given the choices of: Very Satisfied, Satisfied or Unsatisfied, which would you pick to describe the satisfaction levels associated with chiropractic referrals at the Hub?*
  *Prompt: Why is that?*
10. Would you seek chiropractic care for yourself at the Hub or elsewhere?
  *Prompt: Why or Why Not?*
11. What factors would influence your decision to seek chiropractic care for yourself?
  *Prompt: Why or Why Not?*

*Invite participant to add any additional comments that they wish to share. Advise that if they would like to review or add to their responses to contact interviewer or person on research project information sheet.*

## Appendix C. Summary of Responses to Quantitative Questions within Semi-Structured Interviews for Information Purposes

**Satisfaction of chiropractic services delivered to referred patients onsite**					
	Highly Satisfied	Satisfied	Unsatisfied	Did not Refer (due to role) ^1^	Did not Refer (due to choice) ^2^
Timepoint 1	3	4	0	3	1
Timepoint 2	4	3	0	3	1
**Confidence in the quality of chiropractic services delivered to patients onsite**					
	Highly Confident	Confident	Not Confident	No Opinion (due to role) ^1^	No Opinion (no prior exposure)
Timepoint 1	4	6	1	0	0
Timepoint 2	5	5	1	0	0
**Changes in perception towards chiropractic care as treatment modality after working onsite**					
	Improved	Unchanged	Worsened	No Opinion (due to role) ^1^	No Opinion (no prior exposure) ^3^
Timepoint 1	6	4	0	0	1
Timepoint 2	6	5	0	0	0
**Changes in perception towards the chiropractic discipline after working onsite**					
	More Positive	Remains Positive	Neutral	Remains Negative	More Negative
Timepoint 1	6	1	1	3	0
Timepoint 2	4	3	3	1	0
^1^ Respondents who did not refer due to role were in jobs described as medical manager, specialist, nurse, support worker or specialist GP. They described providing advice to patients on how they could self-refer to chiropractic care at specialist reception onsite, and did not view this as type of guidance as a ‘referral’ within their allopathic work environment. ^2^ Respondents who ‘chose not to refer’ to the chiropractor onsite (n = 2) frequently referred to other allied health services onsite. These participants (n = 2) were ‘not confident’ with services delivered and their perception towards chiropractic care was ‘unchanged’ with view towards the chiropractic discipline as ‘remains negative’. ^3^ ‘No opinion, no prior exposure’ were respondents who reported no experiences of chiropractic care personally or professionally prior to working onsite.					
